# Supplementary material for: Are carbon emissions trading and green financial instruments synergistic? -Comprehensive quantitative research based on content analysis
Source: PLoS One. 2024 Mar 7;19(3):e0298601. doi: 10.1371/journal.pone.0298601 (PMC10919601; doi:10.1371/journal.pone.0298601)
Supplement: S1 Data — (ZIP) [file pone.0298601.s001.zip › Data/地级市Z_F工作报告.zip!/╔┬╬≈/╬╝─╧╩╨/└┤╫╘╣1⁄2╓┌║┼í╛┬φ┐╦╩2╛▌═°í┐_2017.docx]

各位代表：

　　现在，我代表市人民政府向大会作工作报告，请予审议，并请市政协委员和列席人员提出意见。

　　一、2016年工作回顾

　　过去一年，面对严峻复杂的经济环境，在市委的坚强领导下，我们认真贯彻中省决策部署，主动适应新常态，积极践行新理念，克难攻坚，稳中求进，全市经济社会保持平稳健康发展的良好态势。生产总值完成1488.6亿元，增长7.5%；全社会固定资产投资完成2289.5亿元，增长10.6%；地方财政收入完成65.7亿元，同口径增长10.6%；社会消费品零售总额达到574亿元，增长14.1%；城乡居民收入达到27485元和9415元，分别增长7.9%和8.2%，较好完成了市五届人大一次会议确定的目标任务，实现了“十三五”平稳起步、良好开局。

　　项目投资支撑有力，经济实现稳中有升。坚持把扩大有效投资作为促发展的第一抓手，出台工业促投资稳增长九方面措施，设立总规模376亿元的产业投资基金，精心策划123个PPP项目纳入中省项目库。坚持包联包抓、一线督办，有力促进了项目落实。沿黄公路渭南段全线贯通，中垦乳业等35个项目建成运营，富平热电联产等115个项目超额完成年度任务，国道310改扩建等80个项目加快推进，韩合机场等30个项目提前开工，316个市级重点项目完成年度投资任务的118.2%。坚持领导带头，主动出击，招大引强，民机试飞基地、中心城市碧桂园等项目成功落地，全年签约招商引资合同396个，到位资金675亿元，加快发展的基础更加牢固。

　　产业转型升级提速，发展动能持续增强。下大气力调结构、促转型，着力培育新的增长点。加快淘汰落后产能，关闭煤矿18处、退出产能1132万吨，压减炼铁产能60万吨。不断优化工业结构，蒲城清洁能源大型煤制烯烃顺利投产，化工、装备制造、食品工业产值均实现两位数增长。沃特玛、天臣动力电池批量生产，桂花能源、智远光伏并网发电，“中国酵素城”等项目加速推进，3D打印产业化初见成效，战略性新兴产业增加值增长11.4%，拉动工业增长新的动能已经显现。园区创建步伐加快，大荔科技园成功晋升省级经济技术开发区。大力发展现代农业，粮食总产达到211万吨，省级农业园区发展到48个，市级以上龙头企业达到428家，新增农民专业合作社454家。白水苹果、蒲城酥梨、大荔冬枣、临渭葡萄、合阳红提等五大公用品牌价值日益凸显，我市荣获“全国果品品牌创建示范市”称号。做大做强现代服务业，华山景区入选“中国十大生态旅游景区”，卤阳湖、党家村成功创建4A级景区，乡村游、休闲游蓬勃发展，全年接待游客4800万人次，旅游综合收入366.3亿元、增长13.1%。积极培育新业态，富平县、大荔县被列为全国电子商务进农村示范县，全市电子商务企业达到248家。成功举办第26届中国厨师节，同州里、中华郡等一批特色街区建成投用，“体育+文化+旅游”成为经济增长新亮点。

　　新型城镇化步伐加快，城乡面貌焕然一新。围绕做大中心城市、做强县城、做美镇村，高起点修编了新一轮城市总体规划。中心城市138个重点城建项目完成投资141亿元，渭东新城全面启动，城区13条“断头路”相继打通，城市框架不断拉大。连霍高速东入口改造竣工通车，渭南北水厂一期建设完成，人民公园、湭河湿地公园加快建设，乐天大街、人和片区成为中心城市新的风景线。市博物馆、图书馆全面开放，5所新建学校顺利招生，全国文明城市通过年度测评，城市品位不断提升。韩城黄河沿岸区域中心城市初步形成，蒲城撤县设市稳步推进，庄里镇综合改革试验区挂牌运行，省级园林县城实现全覆盖，合阳县率先创建国家园林县城。26个省市重点示范镇、文化旅游名镇完成投资40.4亿元，设施功能不断完善。新改造棚户区2.78万套，全面放开落户限制，6.46万人进城落户，城镇化率达到44.07%。

　　环境治理力度加大，生态建设成效明显。坚持“铁腕治污”，狠抓治水绿化，打造生态宜居环境。强力推进“治污降霾·保卫蓝天”行动，集中整治环保突出问题，全市拆改燃煤锅炉407台，淘汰黄标车、老旧机动车1.67万辆。持续开展新一轮渭河治理三年行动，渭河出境水质达到省考目标。电力、化工等重点企业超低排放、脱硫脱硝和除尘改造全面完成，主要污染物减排如期达标，单位GDP能耗下降3.73%。非法采石得到遏制，秦岭北麓和桥山生态修复稳步推进。实施造林绿化63.9万亩，治理水土流失面积551平方公里。编制渭南水系规划，启动渭河生态区建设和黄河西岸综合治理，卤阳湖生态修复和二华蓄滞洪区工程完成节点任务。合阳县荣获国家美丽乡村建设示范县，全市5个镇、15个村被评为省级生态镇村，美丽乡村建设成为全省标杆。

　　改革开放深入推进，发展活力快速释放。持续扩大改革开放，以创新理念引领发展。在西安设立渭南科技工作站，构建“西安研发、渭南制造”新模式。创建国家级“众创空间”2个，市级“众创空间”达到15个，韩城市、大荔县、白水县成为全省县域创新试点县。深入推进七大领域重点改革，103 项年度改革任务顺利完成。“放管服”改革不断深化，取消、下放市级行政许可事项92项，市县两级权责清单向社会公开，镇村便民服务机构覆盖率分别达到93.7%和84%，群众办事更加便捷。商事制度改革实现新突破，催生市场主体3.9万户，非公有制经济比重达到49.4%。新一轮国企国资改革扎实推进，市产投集团国有资本投资运营改革顺利启动。财政管理体制改革持续深化，市级财政专项资金“拨改投”全面实施，与省金控、陕西能源公司基金合作项目正式运行。农村土地确权登记颁证工作率先完成。主动融入“一带一路”，与俄罗斯阿穆尔共青城缔结友好城市，新增外贸备案企业43户，实现进出口总额12.1亿元。晋陕豫黄河金三角区域合作、西渭融合更加紧密，对外开放迈出新步伐。

　　民生保障更加有力，群众得到更多实惠。全年财政民生支出299.56亿元，“两个80%”全面落实。全力推进脱贫攻坚，投入扶贫资金75亿元，搬迁困难群众2.75万户、10.3万人，359个贫困村、26.12万人实现脱贫。城镇新增就业6.4万人，农村劳动力转移就业105.4万人。全面落实普通高中免费教育，创建标准化学校165所，新建公办幼儿园81所，潼关县、华阴市、白水县义务教育发展基本均衡通过国家验收。稳步推进医药卫生体制改革，优化调整公立医院医疗服务价格，56个县级公立医院市县医疗联合体实现全覆盖。成功承办第十一届中国艺术节，秦腔现代剧《家园》获特别贡献奖，我市荣获“国家公共文化服务体系示范区”称号。举办了国际马拉松赛、百城自行车赛等重大赛事，带动体育文化、体育产业快速发展。举行了全国青少年阳光体育大会、全民健身系列活动，群众参与体育运动积极性持续高涨。双拥共建再获殊荣，实现全国双拥模范城“五连冠”。平安渭南、法治渭南建设稳步推进，安全生产形势平稳，有效化解了一批矛盾纠纷和信访积案，群众安全感和满意度进一步提升。

　　切实转变工作作风，自身建设不断加强。认真贯彻党的十八届五中、六中全会精神，全面落实从严治党要求和省委“三项机制”，扎实开展“两学一做”学习教育，强化审计监督和效能监察，谋事干事氛围更加浓厚。法治政府建设进一步深化，决策机制不断完善。自觉接受人大法律监督和政协民主监督，人大代表建议、政协委员提案全部办结。持续推进政务公开，向人民报告、百姓问政、邀请公民进政府活动实现常态化。深入推进政府系统党风廉洁建设和反腐败工作，查处各类违纪案件410件，给予纪律处分476人。

　　国防动员、国家安全、人民防空、民族宗教、外事侨务不断加强，统计、气象、地震、档案、地方志、新闻出版和广播电视等各项工作取得新成绩，妇女儿童、老龄、残疾人、红十字事业都有新进步。

　　各位代表，过去一年，我们顶住了经济下行的压力，经受了去产能的阵痛，保持了经济社会平稳较快发展。取得这样的成绩，是省委、省政府和市委坚强领导的结果，是市人大、市政协监督支持的结果，更是全市广大干部群众同心协力、团结拼搏的结果。在此，我代表市人民政府，向全市广大干部群众、驻渭部队指战员、武警官兵和公安干警致以崇高的敬意！向给予政府工作大力支持的人大代表、政协委员、离退休老同志和社会各界人士，表示衷心的感谢!

　　在肯定成绩的同时，我们也清醒地认识到，渭南发展中还存在不少困难和问题。主要是：转型升级任务艰巨，加快发展压力较大；民间投资活力不强，营商环境还需优化；环保问题比较突出，生态建设亟待加强；改善民生与群众期盼还有差距；一些政府工作人员担当意识、主动作为还不够强。我们将直面问题，细化措施，认真加以解决，推动各项工作再上新台阶。

　　二、今年的主要目标和任务

　　2017年是推进供给侧结构性改革的深化之年，也是贯彻落实市五次党代会精神的开局之年。面对新形势、新要求，我们要抢抓“一带一路”、新一轮西部大开发、关中协同创新的新机遇，突出重点，抓住关键，坚决打好四化同步、治污降霾、环境优化、扶贫脱贫和壮大民营经济五大攻坚战，以勇于担当、敢为人先的精气神，争当全省追赶超越排头兵。

　　今年政府工作的总体要求是：全面贯彻党中央各项决策部署和习近平总书记系列重要讲话精神，紧紧围绕“五位一体”总体布局和“四个全面”战略布局，坚持稳中求进工作总基调，积极践行新发展理念，以贯彻落实市五次党代会精神为统揽，以追赶超越为主题，以转型升级为主线，加快“三地一中心”建设，促进全市经济社会高质量可持续全面发展，以优异成绩迎接党的十九大和省第十三次党代会胜利召开。

　　全市经济社会发展的主要预期目标是：生产总值增长9%左右；全社会固定资产投资增长12%以上；地方财政收入同口径增长6.8%；城镇调查失业率控制在4%以内；城乡居民收入增长10%和11%；万元GDP能耗下降3.5%。这些目标的确定，充分考虑了经济发展新特征、稳中求进总基调，与我市“十三五”规划目标相衔接，既符合加快发展、追赶超越的要求，也有利于推动转型升级、改善民生。我们要以“跳起来摘桃子”的进取精神，努力在实际工作中争取更好结果。

　　实现上述目标，必须坚持追赶超越主题，按照引领黄河金三角、高于全省水平的目标定位，在项目建设、改革创新诸多方面、各个行业，紧盯先进，奋力赶超，力争各项工作走在全省、全国前列；必须坚持转型升级主线，实施五大产业转型发展规划，着力化解煤炭等过剩产能，推动一二三产融合发展，使产业结构紧跟需求变化，让新兴产业成为引领发展的新动能；必须坚持市域统筹发展，牢固树立全市“一盘棋”思想，促进产业统一布局、基础设施统筹规划、公共服务共建共享，加快构建县域错位发展、协同协作、互促共动新格局；必须坚持改革创新引领，进一步解放思想，以改革破难题、添活力，全力推进区域协同创新、深度融合，大力营造鼓励创新、支持创业的浓厚氛围，让改革创新真正成为经济增长的强劲引擎。

　　今年重点抓好八方面工作：

　　（一）全力扩大投资，夯实发展基础

　　把项目建设作为追赶超越的关键来抓，千方百计扩大有效投资，确保固定资产投资增长13%。

　　加快推进项目建设。全年实施市级重点项目330个，投资突破950亿元。建成韩合机场、韦罗高速等50个项目，完成蒲白高速路基桥梁工程，推动西延高铁富平段、西韩城际铁路、合阳-铜川高速、华山机场等项目建设，加快临潼-华山、临渭-蒲城和主城区轨道交通等项目前期工作。持续实施灌区续建配套节水改造、水保治理等水利项目，加强供水、供电、供气等基础设施建设。聚焦产业类项目，确保安泰科技等30个项目建成投产，帝亚一维纯电动汽车等24个项目开工建设。进一步优化投资发展环境，为项目建设提供有力保障。

　　重视项目谋划储备。加大项目策划投入，借助专业机构开展项目策划包装，推动项目由数量扩张向质量提升转变。围绕精细化工、装备制造、食品医药、新能源新材料、航天航空五大产业，精心谋划一批具有引领作用的新兴项目和产业配套项目，全年策划重大前期项目200个以上。加强与中省部门沟通衔接，争取更多项目纳入中省盘子。积极推行PPP等投融资模式，在基础设施、公共服务领域推出一批适合民间投资的重大项目。

　　提升招商引资水平。制定出台全市招商引资优惠政策，进一步夯实领导干部招商责任，推动招商引资实现新突破。聚焦高科技、战略性新兴产业，紧盯500强企业、大型央企民企，实施产业链招商、产权招商、股权招商和轻资产招商，着力引进一批技术先进、带动力强、附加值高的项目。充分发挥驻外联络机构、民间商会和西安科技工作站作用，强化宣传推介，吸引更多企业来渭投资。积极参加丝博会等国内外大型展会，主动参与央企、京企进陕对接，狠抓签约项目落地，确保招商引资到位资金达到710亿元。

　　（二）深化供给侧结构性改革，加快工业转型升级

　　以建设新型工业聚集地为目标，着力去产能、促转型、育新兴，新增规上工业企业50户，规上工业增加值增长8%以上。

　　改造提升传统产业。运用市场化和法治手段，稳妥推进去产能工作，淘汰煤炭过剩产能279万吨，关闭整改无望的水泥企业。加快在建煤矿企业产能释放，建成富平热电联产项目，推进蒲城清洁能源二期、渭南热电联产项目建设，实现能源高端转化和综合利用。开展“中国制造2025”渭南行动，加快龙钢转型升级，加大金钼、中金等企业技改力度，重点实施年产100万吨带钢生产线、陕焦扩能技改等项目，支持中联重科、北人印机等骨干企业转型升级，以新技术、新业态提升装备制造业水平。强化“中国酵素城”引领作用，加快大荔、富平、蒲城、白水食品产业园建设，带动食品工业快速发展。

　　大力发展新兴产业。抓好渭南高新区新能源产业园建设，加快沃特玛二期、天臣三期等项目进度，打造西部重要的新能源动力电池生产基地。实施光伏领跑者计划，建成拓日新能等光伏电站，不断扩大装机规模。加快卤阳湖民机试飞基地、韩城通航产业园建设，促使航空产业取得突破性进展。积极培育生物医药、电子信息等新兴产业，壮大3D打印、聚泰功能新材料等产业集群，力争新兴产业增加值增长12%以上。

　　推进园区提档升级。完善园区基础设施和功能配套，支持民营资本开发工业地产，加快标准厂房和服务平台建设，年内各园区建成1个中小微企业孵化基地。着力提升产业聚集水平，每个园区建成1个百亿元产业集群、1家投资10亿元以上龙头企业，进一步发挥园区支撑引领作用。支持渭南高新区建设，强化体制、管理、服务和模式创新，拓宽发展空间。推进渭南经开区、韩城经开区晋升国家级经开区，澄城工业园晋升省级经开区，富平、蒲城高新区和大荔经开区快速发展，临渭区双创基地、华阴医药产业园加快建设，力争每个县区都有一个省级园区。

　　扎实做好“双创”工作。积极申报国家创新型城市试点，实施创新争先行动计划。鼓励企业与高校院所建立产业技术创新联盟，引进先进技术，开展成果转化，新建工程技术（研究）中心5个，新增高新技术企业5家。利用现有科研用房、闲置厂房、办公楼宇等资源，鼓励招引科技型企业和研发运营机构入驻，新增一批“众创空间”。完善落实相关扶持政策，支持返乡人员、大学生团队和高技能人才创新创业。全年技术合同交易额实现1.8亿元，专利申请和授权分别达到2150件和700件。

　　（三）提高农业供给质量，促进农民持续增收

　　加快规模化、标准化、品牌化建设，推动农业提质增效，培育发展特色农产品，着力打造现代农业示范地。

　　发展壮大现代农业。实施整镇连片现代粮食基地建设，粮食总产稳定在210万吨以上。加大土地流转力度，扩大特色果菜种植规模，全市水果、瓜菜发展到387万亩和195万亩，新增优质水果15万亩。实施畜牧业振兴计划，加快陕西现代畜牧业示范区建设，新扩建标准化养殖场200个以上。增加绿色优质农产品有效供给，建立健全质量追溯体系、产品评价认证体系，推进五大区域公用品牌建设，力争把“白水苹果”、“大荔冬枣”、“韩城大红袍花椒”打造成具有国际竞争力的特色品牌。推动现代农业园区标准化建设，新增市级以上园区10个，新增园区面积5万亩。

　　促进一二三产融合发展。加大龙头企业培育力度，支持农民专业合作社、家庭农场和专业大户发展，新增新型农业经营主体370个。积极实施产业融合发展“县镇村”示范工程，支持大荔县开展国家农村一二三产融合发展试点。扎实推进“互联网+现代农业”行动，不断提高农产品加工比重，加快旅游观光、苗木花卉产业发展，使农民在全产业链中获得更多收益。

　　深化农村综合改革。全面落实农村土地所有权、承包权、经营权“三权分置”政策，加快华州区、富平县、白水县、合阳县产权流转交易市场（中心）建设。抓好5个国定贫困县涉农资金整合试点。启动华州区集体产权制度改革，稳步推进农村集体经济组织股份合作制改革。推广合阳县“农权贷”改革经验，激发农村发展活力。

　　（四）加快发展现代服务业，实现消费较快增长

　　出台加快现代服务业发展的意见，支持新业态发展，确保服务业增加值占GDP比重达到40%以上。

　　大力发展旅游产业。以建设华夏山水文化旅游目的地为目标，实施景区提升工程，加快司马迁祠、少华山、洽川5A级景区创建步伐，年内建设4A级景区2家、3A级景区5家。积极发展红色游、乡村游和文化游，加快仓颉庙、潼关古城、蒲城槐院里等项目建设。推进资源整合、文化旅游融合，打造“风追司马”、“诗经之源”、“华山论剑”等文化演艺精品。支持华旅集团参与黄河金三角区域景区建设和联合营销，努力将华旅集团发展为区域旅游业龙头。支持韩城市、华阴市、大荔县、合阳县创建国家全域旅游示范区，着力构建特色鲜明、多元化旅游新格局。全年旅游综合收入突破400亿元。

　　培育壮大新型服务业。积极发展融资租赁、研发设计、服务外包等生产性服务业，推动二三产业联动发展。依托“互联网+”新模式，加快电子商务平台和物流配送体系建设，实施经开区铁路物流园等一批物流工程，规划建设区域农产品交易中心，新增大型电子商务企业50户。推进“体育+”模式，重点培育运动休闲、竞技表演、场馆服务等体育产业。加快尧头窑文化旅游生态园、桃花源民俗文化园、莘国水城建设，发展文化创意、设计服务、数字动漫等产业，不断提高文化产业在服务业中的比重。

　　加快消费供给升级。实施“家政社会化服务、生活品质提升、标准化和品牌引领、市场主体培育”四大工程，抓好中心城市建国饭店、宏帆广场等项目建设，完善提升万达、信达、华盛三大商业聚集区，建设“15分钟”社区便民生活服务圈。加强“陕菜之都”品牌建设，打造一批特色街区，支持发展健康养老、教育培训等服务消费，拓展休闲体验、假日经济等新型消费。年内新增限额以上贸易企业60户，全社会消费品零售总额增长12%以上。

　　（五）大力推进新型城镇化，促进市域统筹发展

　　围绕建设黄河金三角区域中心城市，加快补齐城镇短板，增强综合承载力和辐射功能，推动城乡协调发展。

　　提升中心城市功能。实施“拉大框架、补齐短板、提升品位、完善功能、塑造新形象”五大工程，重点抓好202个城建项目，确保完成投资283亿元。加快渭东新城、滨河新区和华州大道建设，推动华州区和中心城区深度融合。启动中心城市环城道路建设，实施“四纵四横”主干道路提升改造工程，打通前进路北段、仓程路南延线、杜化路中段等主干道，提升道路绿化亮化美化水平。加快“引水进城”步伐，年内建成西海、渭清、南湖三大公园。持续完善供热、供水和“地下管廊”等市政设施，推动“数字化城管”平台建设，提升综合承载力。加大旧城改造力度，改造棚户区2.4万套，全面推行货币化安置，发展住房租赁市场，促进房地产健康发展。

　　推动县城扩容提质。支持韩城建设黄河沿岸区域中心城市，推进蒲城撤县设市，增强辐射带动能力。加快富平、大荔、华阴等县城建设，完善基础设施和公共配套，推动产业、人口向县城聚集。突出抓好26个省市重点示范镇、文化旅游名镇、特色小镇建设，培育一批富有特色和活力的现代化城镇。积极推进县城“多规合一”，完善建制镇总体规划和村庄建设规划，每个县年内至少建成5个新型农村社区。

　　统筹城乡协调发展。紧抓中省促进区域协同发展机遇，争取更多内联外引城镇项目和资金。统筹推进富平-韩城、临渭-潼关、黄河西岸城乡统筹示范带建设，提升联动发展水平。全面落实农村转移人口市民化政策，推进“镇改街办”、“村改居”工作，完善农民进城落户配套政策，年内进城落户10万人，城镇化率达到48%。全域建设美丽乡村，优化提升210个示范村，持续改善人居环境。

　　（六）加强环境保护，推进生态文明建设

　　坚持生态优先、绿色发展，实施山水林田湖综合治理，努力建设天蓝地绿水清的美丽渭南。

　　强力推进环境整治。持续开展“治污降霾·保卫蓝天”行动，坚持减煤、控车、抑尘、治源、禁燃、增绿六措并举，加大民用散煤清洁化治理力度，实施地热、光伏等清洁能源替代工程，确保空气质量趋于好转。全面实行河长制，完成新一轮渭河治污三年行动任务，做好重点水源地保护，全市集中式水源地水质达标率100%。加快工业园区、重点镇污水处理厂提标改造，确保渭河出境断面水质稳定达标。实施土壤污染治理与修复，支持潼关县土壤污染综合防治先行区建设。大力发展循环经济和清洁生产，促进资源节约高效利用。

　　加大生态保护力度。严守生态保护红线，推进采煤沉陷区治理和秦岭北麓、桥山生态恢复，坚决制止违规采石和乱批乱建。支持富平县山水林田湖生态保护系统建设试点。扎实做好地质灾害防治，保护湿地资源。坚持最严格的节约集约用地制度，切实保护耕地和基本农田，积极盘活存量土地，加强低效用地再开发。倡导绿色消费，推广低碳生活方式。加大环境执法力度，严厉打击环境违法行为。

　　实施造林绿化和水系建设。深入开展“绿化秦东大地、建设美丽家园”三年行动，抓好中心城市、县城出入口、高铁和公路沿线等重点区域造林绿化，推进黄渭洛沿岸防护林建设，全年新增造林绿化50万亩。坚持柔性治水思路，启动渭南水系建设，加快卤阳湖生态修复、二华蓄滞洪区建设、黄河西岸综合治理进度，实施县城水网、乡村涝池恢复扩建等工程，构建纵横成网、河湖相联、渠库相通的生态水系。

　　（七）着力保障和改善民生，提高群众生活质量

　　持续抓好“两个80%”民生政策的落实，扎实推进脱贫攻坚，提升公共服务水平，不断增强群众获得感、幸福感。

　　抓好精准扶贫精准脱贫。深入推进“五个一批”工程，启动搬迁6.47万贫困人口，抓好1.14万非搬迁贫困人口危房改造。实施设施农业、光伏、电商等产业扶贫，建设一批贫困人口参与度较高的产业基地，落实教育扶贫、医疗救助和社会兜底政策，力争10万人脱贫、260个贫困村退出，蒲城县脱贫摘帽，大荔县、潼关县、华阴市整体脱贫。

　　加强就业和社会保障工作。做好以高校毕业生、复员退伍军人、城镇“零就业”家庭、去产能分流职工为重点的就业工作，新增就业5万人，城镇失业人员再就业1.9万人。实施全民参保计划，完善城镇居民大病保险和重特大疾病医疗救助制度，稳妥做好扩面提标。推进城乡居民医疗保险一体化，实施省内异地就医即时结算和定额结算。加强社会救助体系建设，落实好城乡低保户、五保户等弱势群体社会保障政策，让更多群众享受发展成果。

　　大力发展社会事业。启动第三期学前教育行动计划，年内新建中小学11所、公办幼儿园15所，临渭区、华州区、澄城县通过国家义务教育发展基本均衡验收。支持渭南师院扩大规模，提高办学水平。鼓励社会力量办学，推动职业教育特色化发展。加快“健康渭南”建设，持续深化医药卫生体制改革，落实卫生计生各项基本公共服务和优惠政策。实施全民健身计划，建成镇村体育惠民工程500个，推进县级体育场馆、渭河全民健身长廊建设，承办好国际马拉松赛、陕西首届全民健身大会等重大赛事，推动全民健身与全民健康深度融合。抓好基层综合性文化服务中心建设，办好“青春之梦”等文化惠民活动，提升公共文化服务水平。深化全民国防教育，提升双拥共建质量，推进军民融合发展。

　　提高社会治理水平。全面实施“七五”普法规划，深入推进“平安渭南”建设，夯实安全生产主体责任和监管责任，建立健全风险隐患排查整改长效机制，坚决杜绝重特大事故发生。完善立体化社会治安防控体系，依法严厉打击各类犯罪活动，保障群众生命财产安全。强化食品药品日常监管，确保老百姓餐桌上的安全。切实做好民族宗教工作，维护民族团结、宗教和谐。落实信访工作责任制，依法解决群众诉求，维护社会和谐稳定。

　　（八）深化改革扩大开放，持续增强发展活力

　　坚持把改革开放作为追赶超越的强大动力，加快推进关键性改革，进一步激发社会创造力。

　　深化重点领域改革。稳步推进行政体制和事业单位分类改革，加快城市管理执法体制改革。深化企业登记制度改革，促进市场主体注册简易化。全面推行国有资本投资运营试点，促进混合所有制经济发展。深入推进国家中小城市综合改革，支持韩城计划单列市试点、庄里镇综合改革试验区建设。加强税收征管和非税规范管理，实施财政资金“拨改投”和零基预算管理，提高财政资金使用效益。鼓励支持企业在新三板挂牌，扩大企业直接融资规模。严厉打击非法集资活动，维护规范金融市场秩序。协调推进教育、卫生、社会及生态等领域改革。

　　创新体制机制。大力营造“亲”“清”新型政商关系，放宽民间投资领域和市场准入。推进金融创新，统筹信贷资源配置，加大对重大项目、小微企业和“三农”支持力度，提高金融服务实体经济的质量和效率。强化产业基金、科技创新发展基金等政策扶持，培育一批成长性好的中小企业。支持县域创新发展，鼓励各县突出特色、树立品牌、错位发展，增强县域竞争力。继续推进人才强市工程，完善落实相关政策，支持职业经理人、企业家队伍建设，加快引进和培养一批科技型、高技能人才和高水平创新团队。

　　提升对外开放水平。主动融入“一带一路”建设，积极承接长三角、珠三角及沿海地区产业转移。健全渭南海关大通关联动机制，加快电子口岸建设，鼓励优势企业、特色产品开拓国际市场，打造内陆开放新高地。全力推进晋陕豫黄河金三角区域紧密协作、关中协同创新、西渭加速融合，积极在基础设施互联互通、科技成果转化等领域深化合作，构建全方位对外开放新格局。

　　三、努力建设人民满意政府

　　新的一年，我们将主动适应新形势新要求，不断加强政府自身建设，转变执政理念，推动转型发展、追赶超越，更好地服务人民群众。

　　扎实推进依法行政。进一步强化依法行政能力，严格落实权责清单。坚持重大事项专家论证、风险评估、合法性审查、集体讨论决定等制度，不断提高科学决策水平。规范公正文明执法，严格按照法定权限和程序行使权力。建立完善守信联合激励和失信联合惩戒制度，推进社会诚信建设。自觉接受人大法律监督、政协民主监督和社会舆论监督，认真办理人大代表建议和政协委员提案。

　　努力提升服务水平。持续推进“放管服”改革，进一步精简审批事项，优化办事流程，缩短办结时限。加强市县镇村四级便民服务中心标准化建设，推广“互联网+政务服务”模式，让信息多跑路、群众少跑腿。发扬“马上就办”精神，提升政府工作效能。深化政务公开和政府信息公开，坚持开展百姓问政、向人民报告、公民代表进政府等活动，办好“12345”政府服务热线，提高工作透明度。

　　持续改进政府作风。坚持问计于民、问需于民，深入开展调查研究，集聚群众智慧，合力破解发展难题。弘扬“钉钉子”精神，扑下身子，真抓实干，高效执行中省和市委决策部署，确保各项决策落地生根。强化绩效考核、行政问责，加强效能监察和审计监督，坚决纠正不作为、乱作为和慢作为，让群众切身感受到清风正气。深入落实“三项机制”，鼓励干事，激励探索，全面激发干部干事创业热情。严守党的政治纪律和政治规矩，坚决执行中央八项规定，严格落实党风廉洁建设责任制，严肃查处各类腐败问题，塑造忠诚、干净、担当的政府形象。

　　各位代表、同志们，加快发展正当其时，追赶超越任务艰巨。让我们在省委、省政府和市委的坚强领导下，紧紧依靠全市人民，振奋精神，锐意进取，低调务实不张扬，撸起袖子加油干，为加快建设富裕美丽幸福新渭南不懈奋斗，以优异成绩迎接党的十九大和省第十三次党代会胜利召开！
